# Open vascular treatment of median arcuate ligament syndrome

**DOI:** 10.1186/s12893-017-0289-8

**Published:** 2017-08-29

**Authors:** Mansur Duran, Florian Simon, Neslihan Ertas, Hubert Schelzig, Nikolaos Floros

**Affiliations:** 1 Department of Vascular and Endovascular Surgery, Heinrich-Heine University Medical Center, Düsseldorf, Germany; 2 Klinik für Gefäß- und Endovaskularchirurgie, Universitätsklinikum der Heinrich-Heine Universität, Moorenstr. 5, 40225 Düsseldorf, Germany

**Keywords:** Median arcuate ligament syndrome, Dunbar syndrome, Celiac artery compression syndrome, Celiac artery, Intestinal ischemia

## Abstract

**Background:**

Median arcuate ligament syndrome is a rare condition with abdominal symptoms. Accepted treatment options are open release of median arcuate ligament, laparoscopic release of edian arcuate ligament, robot-assisted release of median arcuate ligament and open vascular treatment. Here we aimed to evaluate the central priority of open vascular therapy in the treatment of median arcuate ligament syndrome.

**Methods:**

We conducted a monocentric retrospective study between January 1996 and June 2016. Thirty-one patients with median arcuate ligament syndrome underwent open vascular surgery, including division of median arcuate ligament in 17 cases, and vascular reconstruction of the celiac artery in 14 cases.

**Results:**

In a 20-year period, 31 patients (*n* = 26 women, *n* = 5 men) were treated with division of median arcuate ligament (*n* = 17) or vascular reconstruction in combination with division of median arcuate ligament (*n* = 14). The mean age of patients was 44.8 ± 15.13 years. The complication rate was 16.1% (*n* = 5). Revisions were performed in 4 cases. The 30-day mortality rate was 0%. The mean in-hospital stay was 10.7 days. Follow-up data were obtained for 30 patients. The mean follow-up period was 52.2 months (range 2–149 months). Patients were grouped into a decompression group (*n* = 17) and revascularisation group (*n* = 13). The estimated Freedom From Symptoms rates were 93.3, 77.8, and 69.1% for the decompression group and 100, 83.3, and 83.3% for the revascularisation group after 12, 24 and 60 months respectively. We found no significant difference in the Freedom From Re-Intervention CA rates of the decompression (100% at 12, 24 and 60 months post-surgery) and revascularisation (100% at 12 months, and 91.7% at 24 and 60 months post-surgery) groups during follow-up (*p* = 0.26).

**Conclusions:**

Open vascular treatment of median arcuate ligament syndrome is a safe, low mortality-risk procedure, with low morbidity rate. Treatment choice depends on the clinical and morphological situation of each patient.

## Background

**Fig. 1:**
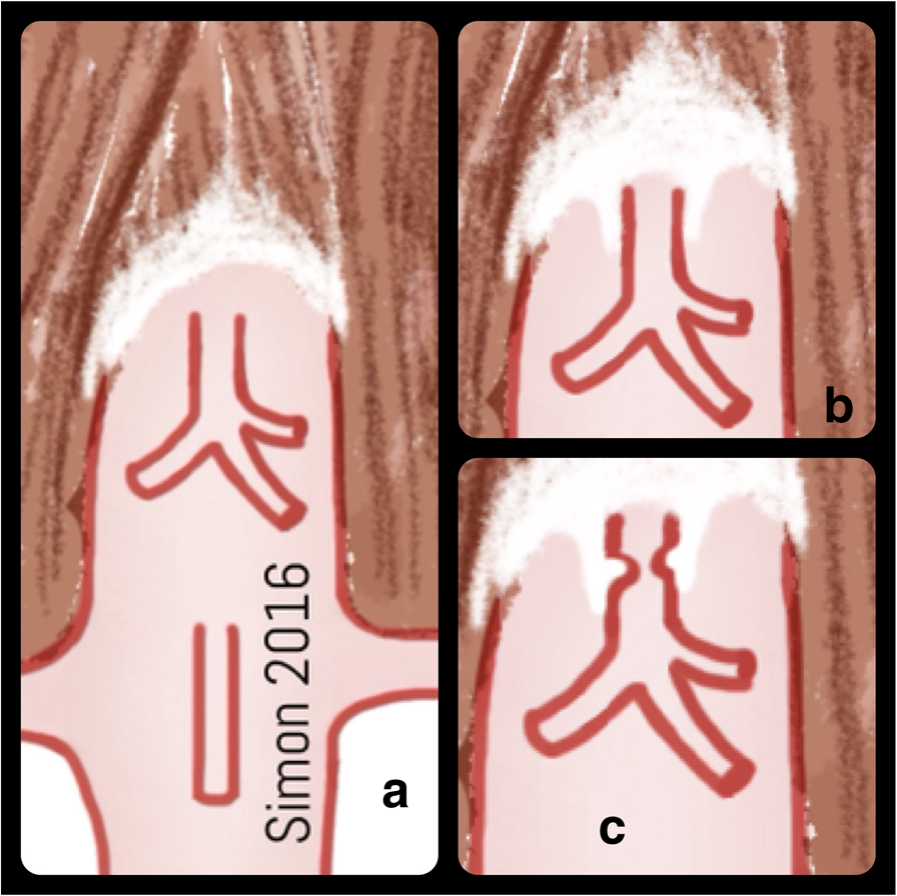
Drawing of MALS. **a** normal anatomy; **b** MAL in inspiration and **c** MAL in expiration with stenosis of celiac artery

Median arcuate ligament syndrome (MALS), also known celiac axis compression syndrome, celiac artery (CA) compression syndrome or Dunbar syndrome, is a rare condition in which the celiac artery is compressed by fibrous bands, the median arcuate ligament, and ganglionic periaortic tissue. The median arcuate ligament (MAL) is a fibrous band of the diaphragmatic crus surrounding the CA. Low insertion of the ligament or high take off of the CA, or both, result in extrinsic compression during expiration (Fig. 1) [1]. MALS occurs in 2 per 100,000 patients [2], often in young women who present with postprandial epigastric pain (80%), nausea (9.7%), weight loss (48%), and diarrhea (7.5%) [3]. CA compression has been found in 34% of autopsies without prior reporting of symptoms. Variability in clinical presentation and unpredictable response to surgery led to scepticism about the clinical significance of some findings [4]. However, newer studies have described significant clinical presentation with positive response to surgery [3]. This study reviewing literature between 1963 and 2012 for MALS (*n* = 400, totally) compared open and laparoscopic surgery. In 85% (*n* = 339) a postoperative symptom relief and in 6,8% for the open and in 5,7% for the laparoscopic group a late recurrence of symptoms were achieved. In 9,1% open conversion were necessary in the laparoscopic group because of bleeding. In the conclusion, the majority of the study population after surgery (open and laparoscopic) symptom relief were achieved [3].

The symptoms associated with MALS have been attributed to visceral ischemia and neurogenic causes, but there is no valid data for this claim [5, 6].

The accepted treatment options for MALS include release of MAL, laparoscopic release of MAL, robot-assisted release of MAL, and open vascular surgery [3, 6, 7]. Endovascular treatment does not solve the problem of extrinsic compression of the CA [6].The objective of our study was to evaluate the long-term outcome after open vascular therapy of MALS using division of MAL and vascular reconstruction of the CA in combination with division of MAL.

## Methods

Between January 1996 and June 2016, 31 patients with MALS underwent a vascular surgery procedure; division of MAL in 17 cases and vascular reconstruction of the CA in combination with division of MAL in 14 cases. Surgical procedure was performed in an open surgery approach in all cases. CA stenosis was defined as a peak systolic velocity > 200 cm/s or end diastolic velocity > 55 cm/s [8]. Diagnosis was provided preoperatively by Angiography in inspiration and expiration conditions. MALS was confirmed intraoperatively after exposure of the CA, revealing extrinsic compression from the MAL, prominent fibrous bands, and ganglionic periaortic tissue, the resection of which released the CA so that it was no longer constricted. In 14 cases the CA had an irreversible fixed stricture or stenosis, so that further vascular reconstruction was needed.

All of the patients treated for MALS at the Department of Vascular and Endovascular Surgery, Heinrich-Heine University Medical Center were identified from a database (*n* = 31). Data were retrospective collected and analyzed. Retrospective data analysis was approved by the review board of the University of Düsseldorf (study number 5617). Patient informed consent was waived because of the retrospective characteristics of the study.

**Fig. 2:**
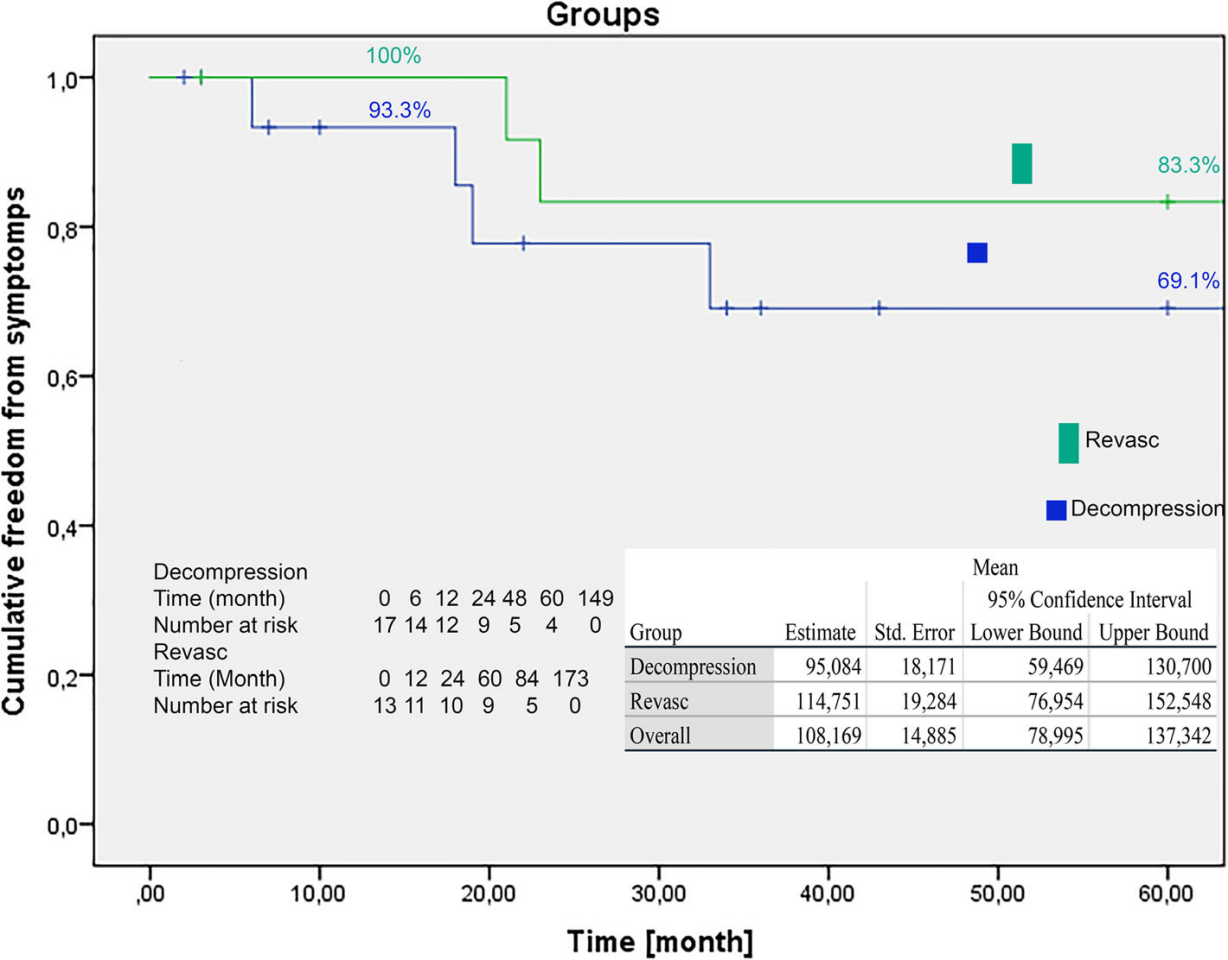
Freedom from symptoms demonstrated in a Kaplan-Meier curve. One- and 5-years rates are presented. Decompression, group treating with division of MAL; Revasc, group treating with vascular reconstruction of the CA in combination with division of MAL (log-rank test: *p* = 0.72)

**Fig. 3:**
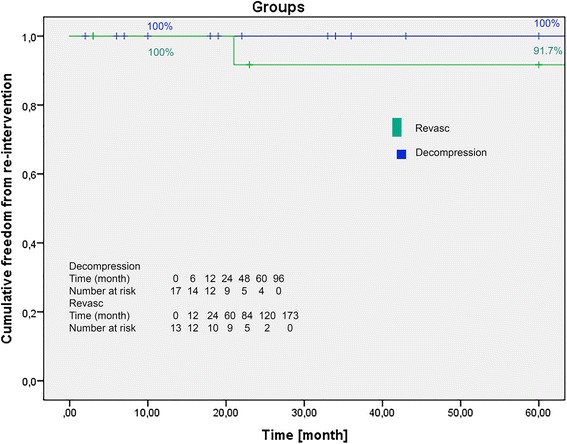
Freedom of re-intervention of celiac artery (CA) demonstrated in a Kaplan-Meier curve. One- and 5-years rates are presented. Decompresion, group treating with division of MAL; Revasc, group treating with vascular reconstruction of the CA in combination with division of MAL (log-rank test: *p* = 0.26)

The primary study end point included freedom from symptoms capturing in a patient questionnaires and freedom from re-intervention using duplex scanning in the follow-up. Patient questionnaires about symptoms and patient history, duplex scanning results, clinical exams were obtained in 30 patients in our outpatient department. The SPSS statistical package (version 22.0) was used for statistical analyses. The results are reported as mean ± standard deviation (SD). Freedom from symptoms (Fig. 2) and freedom from re-intervention (Fig. 3) were calculated with the Kaplan-Meier method and the groups were analysed with the log rank test. Significance was defined as *p* < 0.05.

## Results

In a 20-year period, 31 patients were identified with MALS (*n* = 26 women, *n* = 5 men). These patients were treated with division of MAL (*n* = 17) in an open surgery approach or vascular reconstruction including division of MAL (*n* = 14). The mean age was 44,8 ± 15.13 years (range: 18–68). Patients’ demographic information is summarized in Table 1 and the surgical procedure in Table 2, undergoing an open repair in all cases. In six cases co- surgical procedures were performed because of atherosclerosis of the superior mesenteric artery (SMA) in four cases and of the renal artery (RA) in two cases. These co-procedures were performed only in the first decade of our series, using more aggressive treatment options in this period. The mean body mass index (BMI) of the patients was 21.9 ± 3.27 (range: 16.7–29.2).

**Table 1:** Patient characteristics and co- surgical procedures

Variable	*n*	%
female	26	83.9
Age (mean ± SD years)	44,8 ± 15.13	
Smoking history	8	25.8
Arterial hypertension	8	25.8
Diabtes mellitus	1	3.2
Coronary heart disease	1	3.2
Peripheral vascular disease	1	3.2
Hyperlipidemia	2	6.3
Co- surgical procedures	6	19.4
Superior mesenteric artery:	4	
• SMA transposition	1	
• Transaortic removal of a stent in the SMA	1	
• Patchplasty of the SMA with vein	1	
• Aorto-mesenteric loop-bypass	1	
Renal artery:	2	
• Transaortic thromboendarterectomy of the RA	1	
• Patchplasty of the RA with vein (multiple occurrences possible)	1	

Data are shown as mean±SD for ratio scale data and as frequency distribution with percentages for nominal scale data

**Table 2:** Surgical procedures

Surgical procedure	*n* = 31 (*n*)	%
Decompression of CA	17	65
Decompresssion of CA with vaskular therapie	14	45
➢ Aorto-celiac vein interposition	6	
➢ Aorto-hepatic vein interposition	1	
➢ Resection of the CA and end-to-end anastomosis		
• Due to stenosis	1	
• Due to aneurysm	1	
➢ Transaortic removal of a stent of the CA	2	
➢ Patchplasty of the CA with vein	1	
➢ Transposition of lienal artery	2	

Data are shown as frequency distribution and percentages

The most common preoperatively presented symptom was abdominal pain, which was experienced by patients, 19 of which had postprandial abdominal discomfort. Other symptoms were unintentional weight loss (*n* = 9), nausea (*n* = 6), diarrhoea (*n* = 5), and vomiting (*n* = 1).

Relevant blood test results for intestinal ischemia were normal (mean lactate, 0.92 mmol/l [range, 0.5–1.6 mmol/l]; mean C-reactive protein, 0.90 mg/dl [range 0.00–9.80 mg/dl] and mean leucocytes, 7.32 per nl (range 4.16–16.40 per nl). All patients were admitted to hospital in an elective situation.

Diagnosis was provided by digitally subtracted angiography (DSA) (*n* = 31), computed tomography angiography (CTA) (*n* = 9), magnetic resonance angiography (MRA) (*n* = 8), and duplex ultrasound scan (*n* = 18). Duplex scanning was a part of the evaluation of patients with abdominal symptoms and suspected MAL syndrome.

The complication rate was 16.1% (*n* = 5). Complications consisted of chylous ascites (*n* = 1), wound dehiscence (*n* = 1), transient neurological disorders (*n* = 2), and pleural effusion (*n* = 1) (Table 3). Revisions were performed in four cases: aorto-celiac vein interposition after unsuccessful decompression of the celiac trunk (*n* = 2), re-laparotomy without revealing a visceral malperfusion (*n* = 1), or percutaneous angioplasty (PTA) of the superior mesenteric artery (*n* = 1) (Table 3). The 30-day mortality rate was 0%. Mean in-hospital stay was 10.7 days (range 5–27 days). Post-operative examinations (CTA, DSA, MRA or duplex ultrasound scan) of CA-blood flow were done in all patients before discharge.

**Table 3:** Intra- and postoperative complications

Parameter	*n*	%
Revisions	4	12.9
• Aorto-celiac vein interposition	2	
• Second look laparotomy due to suspected intestinal ischemia	1	
• Angioplasty of SMA bypass due to anastomosis stenosis	1	
Wound healing disorders	1	3.2
Remarkable neurological disorders	2	6.4
• Diplopic images and impaired vision on the left eye	1	
• Distinctive delirium	1	
Chylous ascites	1	3.2
Pleural effusion	1	3.2

Data are shown as frequency distribution and percentages

Follow-up data were obtained for 30 patients using duplex scanning for CA stenosis. One patient lost to follow-up. The mean follow-up period was 52.2 months (range 2–149 months). Patients were grouped in a decompression group (*n* = 17) and a revascularisation group (*n* = 13). The decompression group was treated with decompression of the CA only, while the revascularisation group was treated with decompression and revascularisation of the CA. Overall, freedom from symptoms was described by 20 patients (66.7%) for the follow-up-period, of which 12 (70.6%) belonged to the decompression group and 8 (61.5%) to the revascularisation group. For all patients, the estimated freedom from symptoms rates were 93.3, 77.8, and 69.1% for the decompression group and 100, 83.3, 83.3% for the revascularisation group at 12, 24 and 60 months post-surgery respectively (Fig. 2). The groups were analysed with the log-rank test, showing no significant difference (*p* = 0.72).

Revisions were performed in two cases (PTA of CA [*n* = 1] and multiple revisions [*n* = 1]), both belonging to the revascularisation group. The case with multiple revisions consisted of transposition of the splenic artery in the supravisceral aorta 3 months postoperatively, aorto-CA vein interposition 18 months postoperatively, PTA and stent angioplasty of aorto-CA graft 43 months postoperatively, and aorto-celiac vein interposition 60 months postoperatively. We found no significant difference in the estimated freedom from re-intervention CA rates of the decompression (100% at 12, 24 and 60 months post-surgery) and revascularisation (100% at 12 months, and 91.7% at 24 and 60 months post-surgery) groups during follow-up (log-rank test: *p* = 0.26) (Fig. 3).

## Discussion

The incidence of MALS is 2 per 100,000 patients [2]. MALS is more prevalent in women than men [6, 9]. It regards young patients aged between 30 to 50 [6].

Lipshutz first described the anatomical compression of the celiac artery in 1917 [10] and Harjola described MALS in 1963 [11]. The first MALS clinical study was by Dunbar et al. in 1965 [12]. Since then, many case series and clinical studies addressing MALS have been published. New diagnostic and therapeutic modalities play a significant role in the treatment of MALS. DSA with breathing maneuvers is the standard imaging approach. Duplex abdominal ultrasonography during inspiration and expiration can also be used in the diagnosis of MALS. Gruber (2012) conducted the largest study related to the utility of ultrasound in the diagnosis of MALS. Based on Gruber’s findings, functional ultrasound was recommended as a screening instrument [13]. The CTA and MRA offer a precise 3D visualization of the anatomical structures and are key parts of the routine preoperative examination [14, 15].

In our series, DSA was the preoperative examination used in all cases. Other adjunctive modalities such as the gastric exercise tonometry or percutaneous celiac ganglion block were not utilized.

### Pathophysiology

The pathophysiologic mechanism remains undefined. The existence of celiac compression in asymptomatic patients indicates that there is something more than just the mechanical injury of the vessel caused by extrinsic compression. The increased demand for blood flow after a meal leads to symptoms due to ischemia of the foregut, but an isolated stenosis or even occlusion doesn’t cause such symptoms. Years of experience show that two or more stenosis must be present to cause ischemia due to the extensive collateral circulation [16]. Another theory suggests that a steal phenomenon by larger collateral vessels may cause symptoms in patients with a compressed celiac trunk [1]. There is also a neuropathic approach. Eventually, the compression leads to direct irritation of sympathetic pain fibers, splanchnic vasoconstriction, and ischemia [16]. The high prevalence of asymptomatic patients exhibiting radiographic evidence of celiac compression is the main reason that keeps the pathophysiologic mechanism of MALS unclear.

### Clinical presentation

Although it is difficult to calculate the incidence of MALS in the population, a 10-24% incidence of some degree of radiographic compression is given in literature [17]. The disease is more prevalent in women than in men [6]. In our study the female percentage was 83.9% and a thin body habitus (BMI = 21.9 ± 3.27 with a range of 16.7-29.2). The disease also appears in children. Mak et al. reported a study with 42 patients aged from 8.6 to 20.5 [18].

The majority of the patients in our study showed abdominal symptoms, especially postprandial abdominal pain in 61.3%.

### Management

Treatment modality is important for the morbidity and long-term outcome of MALS treatment. Most recent studies suggest that the laparoscopic approach is best [19, 20]. We believe that an open approach is needed when a structural defect of the vessel is detected or even suspected. Diagnostic tools like the gastric exercise tonometry may help to clarify in vague cases [5].

In our series we performed an open surgery approach and ancillary diagnostic tests were not necessary.

### Open surgery

Since Dunbars first report back in the 1960s, the studies reporting open surgical treatment decreased as minimal invasive surgery emerged. The last large series (*n* = 51) was reported in 1985 by Reilly et al. with good long-term results (77% symptom free after a mean time of 9 years) [21]. In 2013 Sultan et al. reported about 8 out of 11 patients with a complete relief, 1 patient with improvement and 2 patients with worsening of abdominal pain [9]. Our data show good long-term results. The estimated freedom from symptoms rates were 93.3, 77.8, and 69.1% for the decompression group and 100, 83.3, and 83.3% for the revascularisation group at 12, 24 and 60 months after syrgery respectively. The estimated freedom from re-intervention CA rate was 100% (throughout follow-up) for the decompression group, and 100, 91.7, and 91.7% after 12, 24, and 60 months follow-up for the revascularisation group. The statistical analysis shows no significant difference in the long-term outcome for the groups according to clinical symptoms. But nevertheless, the decompression of the CA seems not to be enough, in some cases also an additional revascularisation of the CA is necessary.

### Laparoscopic MAL release

Since the first report on laparoscopic management of MALS from Roayaie et al. in 2000 [22], laparoscopic MAL release tends to be the standard surgical management. The advantages of this approach are clear and include decreased postoperative pain, shorter hospital stay and faster recovery, and decreased postoperative adhesions. In 2016, Weber et al. conducted a large study enrolling 39 patients treated with laparoscopic MAL release, reporting good results in the follow-up (84.6% symptom relief and 10.3% conversion to open surgery due to intraoperative hemorrhage) [19]. An interesting point of this study of Weber et al. is that 6 out of 26 patients during follow-up showed persistent stenosis and 1 occlusion, but were symptom free. This finding suggests an important role of the plexus fibers and raises some interesting questions. For example, is the release of the plexus fibers in some cases sufficient to relieve symptoms, how can we define these cases, and what are the best diagnostic approaches? Further studies will be needed to address these questions. In a recent study Tracci et al. found that MAL release alone could provide relief in a significant proportion of patients. These patients were more likely to have symptom recurrence than those who underwent some form of revascularization [17]. These findings support the contention that residual stenosis should be considered as a treatment option.

In summary, MAL syndrome is a multifactorial disease caused by chronic external pressure on the nearby vessel wall and neuronal structures.

Limitations of our study include the retrospective character and the reliance on patients reports of pain relief.

## Conclusions

Based on our data, open surgery of MALS can be considered safe procedure. Further vascular procedures regarding the specific pathology with fixed stenosis of the CA were needed after decompression. MALS treatment choice should be made based on the clinical and morphological situation of the patient.

## Abbreviations

BMI: Body mass index
CA: Celiac artery
CTA: Computed tomography angiography
DSA: Digitally subtracted angiography
Fig: Figure
MAL: Median arcuate ligament
MALS: Median arcuate ligament syndrome
MRA: Magnetic resonance angiography
PTA: Percutaneous angioplasty
RA: Renal artery
SD: Standard deviation
SMA: Superior mesenteric artery
